# Number of Attributed Reasons for Everyday Discrimination and Mortality Among Older Blacks

**DOI:** 10.1093/geroni/igab046.720

**Published:** 2021-12-17

**Authors:** Ryon Cobb

**Affiliations:** University of Georgia, Athens, Georgia, United States

## Abstract

To date, little is known about the significance of the number of attributions for everyday discrimination on all-cause mortality risk among older Blacks. Data are from a subsample of older Black respondents in the Health and Retirement Study (HRS), a nationally representative panel study of adults above the age of 50 in the 2006/2008 HRS waves, respondents completed a battery of questions on experience with psychosocial stressors, which included the number of attributed reasons for everyday discrimination. Vital status was obtained from the National Death Index and reports from key household informants (spanning 2006–2016). Cox proportional hazard models were used to estimate the risk of mortality. During the 10-year observation period, 450 deaths occurred. A higher number of attributed reasons for everyday discrimination was associated with a higher likelihood of death after adjusting for demographic characteristics and remained significant after further adjustments for other psychosocial, health, behavioral, and economic covariates.

